# Chemical evidence for milk, meat, and marine resource processing in Later Stone Age pots from Namaqualand, South Africa

**DOI:** 10.1038/s41598-023-28577-1

**Published:** 2023-01-30

**Authors:** Courtneay Hopper, Julie Dunne, Genevieve Dewar, Richard P. Evershed

**Affiliations:** 1 Department of Anthropology, University of Toronto Scarborough, 1265 Military Trail, Toronto, ON M1C 1A Canada; 2 School of Environment, University of Toronto, Toronto, Canada; 3 Organic Geochemistry Unit, School of Chemistry, University of Bristol, Bristol, UK; 4 Rock Art Research Institute, School of Geography, Archaeology, and Environmental Studies, University of Witwatersrand, Johannesburg, South Africa

**Keywords:** Archaeology, Lipids

## Abstract

The subsistence practices of Later Stone Age (LSA) foragers and herders living in Namaqualand South Africa are often difficult to differentiate based on their archaeological signatures but characterizing their dietary choices is vital to understand the economic importance of domesticates. However, ethnohistoric accounts have provided information on the cooking/boiling of marine mammal fat, mutton, plants, and milk by early herders and foragers across the Western Cape. To further investigate these reports, we use lipid residue analysis to characterize 106 potsherds from four open-air LSA sites, spanning in time from the early first millennium to the late second millennium AD. Two sites (SK2005/057A, SK2006/026) are located on the Atlantic coast whereas sites Jakkalsberg K and Jakkalsberg M are located further inland on the southern bank of the Orange River. Notably, at the coastal sites, the presence of marine biomarkers suggests the intensive and/or specialized processing of marine products in many vessels. The dominance of ruminant carcass products at inland sites and probable sheep remains confirms the importance of stockkeeping. Furthermore, and in good agreement with ethnohistoric accounts for its use, our results provide the first direct chemical evidence for the use of dairy products in LSA western South Africa.

## Introduction

During the 15th Century, European ships rounding the Cape of Good Hope encountered pastoralists with large herds of livestock^1–4^ at the newly established provisioning station at Cape Town. Yet these sheep and cattle remain archaeologically enigmatic^5^, making it difficult to study Indigenous herd management strategies and human-animal relationships. Questions as to the introduction of domesticated animals to southern Africa, the subsequent spread of herding and the beginnings of dairying practices, also remain unresolved.

**Figure 1:**
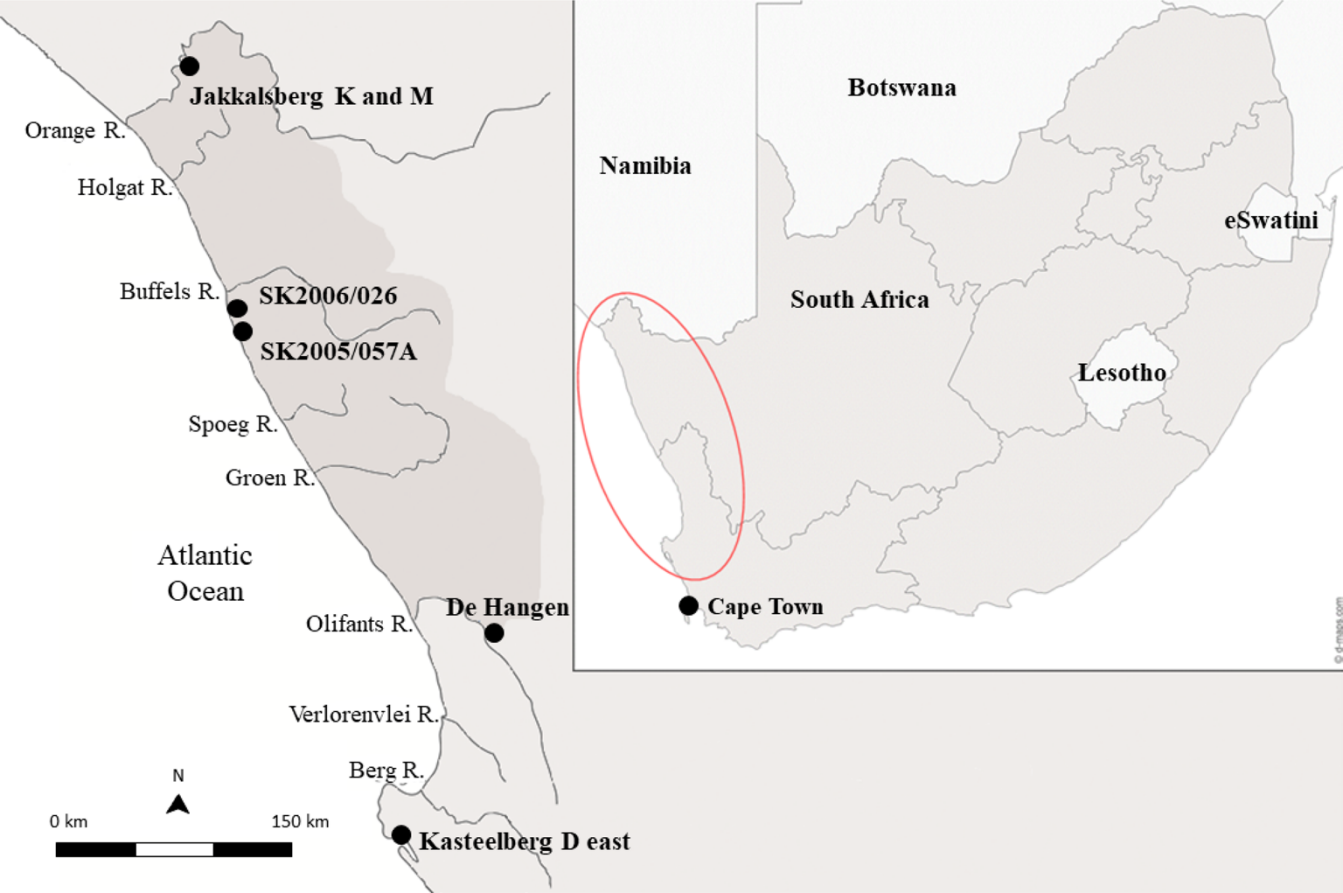
Map of Southern Africa (inset) with Namaqualand enclosed in the darker grey area with approximate location of the archaeological sites mentioned in this study noted. Inset map adapted from https://d-maps.com/carte.php?num_car=4412&lang=en using Adobe Illustrator v. 27.1 (https://www.adobe.com/). Enlarged map adapted using Google Maps (https://www.google.com/maps) in Adobe Illustrator v.27.1 (https://www.adobe.com/).

Looking to the northwest corner of South Africa (Fig. 1), the Namaqualand coastal desert, extending along the west coast over 1000 km, is the optimal location for unequivocal herder sites (indicated by the presence of domestic animal bone) as the homeland of Nama pastoralists. Spoegrivier Cave in Namaqualand is the location of the oldest directly dated (AMS ^14^C) sheep bone (confirmed through palaeoproteomics^6,7^) in South Africa at 2105 ± 65 BP (OxA-3862), together with a horn core from site KN2005/0041 directly dated to 1625 ± 25 BP (OxA-22933) and identified through aDNA as *Bos taurus*^8^, suggesting the early presence of herders in the region. However, although there are many (*c*. 1500) Late Stone Age (LSA: 40 kya to historic times) open-air, single occupation sites in the region (the vast majority being shell middens and scatters along the coastal plain)^9^, to date, only ~ 15 have definitively been identified as herder sites, based on the presence of domesticated animal remains^10^.

One of the difficulties lies in distinguishing between forager and herder sites as both have similar material cultural signatures after 2000 BP: stone tools, pottery, wild fauna, and ostrich eggshell beads^11^. This means that LSA sites with domesticate remains are simply labelled herder and those without, forager. In western South Africa, this oversimplistic dichotomy can easily overlook the fluidity of subsistence practices (e.g., ‘hunter-herders’^12–15^) during the LSA and how groups may have incorporated available resources into their diet. Further complicating matters is that faunal assemblages within the region are often highly fragmentary, making attributions between sheep and similar-sized local wild bovids, such as springbok and grey duiker, somewhat problematic^7,16–18^. This is particularly relevant as the timing of the introduction of domesticated sheep to southern Africa is still not fully understood.

### Ethnohistoric accounts of the use of pottery in western South Africa

**Figure 2:**
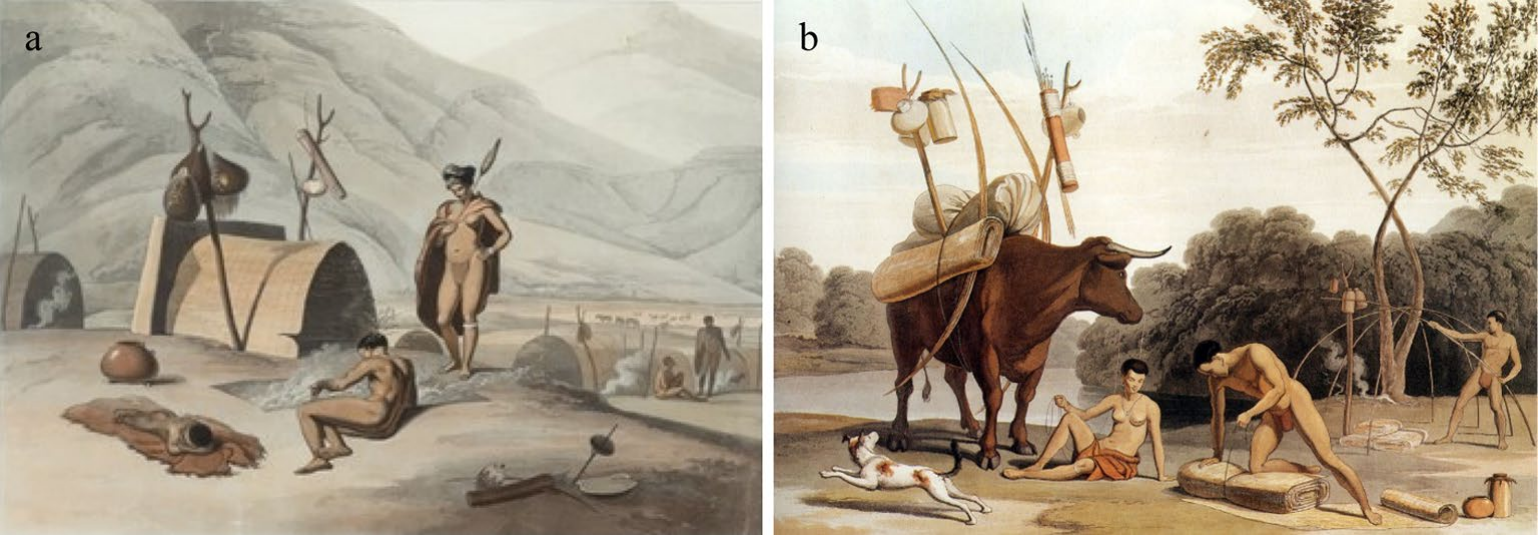
(**a**) ‘*Bosjesmans Frying Locusts’* painted by Samuel Daniell depicting a forager camp with a lugged ceramic vessel. (**b**) ‘*Korah Preparing to Remove*’ painted by Samuel Daniell depicting a “forager-type” bowl in a herder camp being prepared for moving. Distributed under a CC-BY 2.0 license.

Cape Ceramics found along the west coast of South Africa take three broad forms, lugged, spouted, and bowl-shaped. Lugged pots, commonly associated with herders, have pointed bases, flared shoulders, constricted necks and two opposed suspension lugs^12,13,19^. Spouted pots are generally indistinguishable from lugged pottery below the shoulder but have an obliquely angled spout^13^. Bowls are small hemispherical vessels with thick, crudely finished walls and round bases that lack decoration, bosses, or lugs^19^ and are commonly associated with foragers. However, whilst most ethnohistoric accounts^20,^ as cited in ^21^, illustrations^22^ and archaeological^23^ evidence suggest that pottery typology did follow the generalized “herder-forager” dichotomy, there are also accounts of foragers using lugged pottery and herders using open-mouthed bowls. For example, two historical illustrations attributed to S. Daniell^21^ (Fig. 2) show an unmistakeable “herder” lugged vessel standing in a forager camp and the other an unmistakable “forager” bowl hanging from a forked stick strapped to an ox ready for transport^21^ (see also Stewart^24^ for an archaeological example of this fluidity in pottery use).

Although relatively scarce, ethnohistoric accounts have provided some information on what foodstuffs early herders and foragers processed in their ceramic pots, namely the cooking/boiling of marine mammal fat, mutton, plants, and milk^25^. For example, in 1654, Jan van Riebeek (of the Dutch East India Company) noted that he observed Khoekhoe (non-Bantu speaking Indigenous nomadic pastoralists) using pottery to render whale blubber^26^. Certainly, post-contact herders were known to have used marine mammal grease as an important component of body adornment^26^ and as a supplement to their lean diet^27^.

In the course of his travels through the Western Cape in the late 1700’s, Le Vaillant noted that pottery appeared to be used for the purpose of melting animal fat into grease and discusses Khoekhoen guides breaking up elephant bones and rendering their grease in tea kettles^28^. Laidler^10^, during his travels through Namaqualand in the early 1900’s, observed that pots were of such hardness that they could be used for rendering fat^29^. However, there are relatively few accounts specifying which animals were processed, although Laidler^10^ notes that when a springbok was killed, its blood was brought home in its own stomach and boiled in a new pot, which would then be used to cook the meat from the springbok^29^. There are also a few (vague) accounts of ceramic pots being used to cook the meat of domesticates (cattle, sheep) and/or wild game in their own fat^30,31^.

Although ethnohistoric accounts reveal that milk comprised an important component of the diet of herding peoples living in western South Africa^1,32–34^ they generally suggest that milk was rarely, if ever, stored in pots^25^. Instead milk would either be consumed directly from the animal or collected in organic containers such as wooden buckets, calabashes, reed baskets, and leather bags^25,35^. Despite this, there are some reports of milk being cooked/boiled with meat and/or plants in ceramic pottery for both subsistence and medicinal uses. In 1731, during his travels through the south western Cape, Kolb^36^ noted the cooking of mutton, the entrails of cattle, and wild game with blood and milk, while, in 1907, Schultze^35^, travelling through Namaqualand and the Kalahari, discussed the consumption of game hide that was first boiled for an extended period in milk. He also noted that the Khoekhoen would sometimes boil bulbs, corms, or blossoms from *Gazania* spp. (Namaqualand daisy) with milk to make a porridge. They would also raid ant nests for buffalo grass (*Aristida* spp.) which they roasted, ground to flour, and cooked with milk into a porridge.

Schultze^19^ also described medicinal remedies in which milk was used. In particular, the treatment for chickenpox was to drink diluted milk boiled with a handful of goat’s dung daily until symptoms receded^25^. Watt and Breyer-Brandwijk^37^ also recorded the Nama boiling the tubers of *Pelargonium antidysentericum* in milk for the treatment of diarrhea. Milk was also used as a topical treatment. For example, Dapper^30^ observed healers, after cauterizing lacerations, applying freshly-cooked sweet milk mixed with a specific herb (species not mentioned) to the wound.

### Previous organic residue analysis in Southern Africa

Early research using organic residue analysis attempting to identify herding and, by extension, the exploitation of milk products, in western South Africa, comprises two published studies^38–40^. Neither yielded evidence for dairy processing although it should be noted both were very small studies. An early study by Patrick et al.^38^ analyzed charred material from the inner walls of potsherds from two sites: Kasteelberg B, a coastal herder site, and Die Hangen, an inland forager site (Fig. 1). The residue from one Kasteelberg B vessel contained marine lipids (thought to be seal, based on the large numbers of their bones at the site), whereas (a small number of) potsherds from De Hangen, revealed terrestrial animal fats, although these could not be attributed to source.

More recently, potsherds from putative specialised vessels known as ‘spouted ware’, found at Kasteelberg D east (Kasteelberg De) in contexts containing high proportions of sheep bones and thus thought to have been used for storing and pouring sheep’s milk, were analysed. However, these vessels, from part of the same herder site complex as Kasteelberg B^39^, were predominantly used to process marine-derived animal products, despite sheep bones being abundant at the site. In contrast, in the highlands of Lesotho, lipid analysis of hunter-gatherer pottery from the sites of Likoaeng and Sehonghong indicated that one-third of vessels were used to process dairy products^40^. Notably, compound-specific lipid dating revealed these dated from the mid and late first millennium AD, respectively.

Consequently, as questions regarding the identification of herders and the exploitation of milk and milk products in western South Africa remain unresolved, here we carry out a large-scale study using organic residue analysis of potsherds from four archaeological sites located in Namaqualand to further investigate this issue. In recent decades, lipid residue analysis of archaeological pottery has become a core tool for exploring ancient diet. The use of gas chromatography (GC), gas chromatography–mass spectrometry (GC–MS) and compound-specific stable carbon isotope analysis allows differentiation between carcass fats from ruminant and non-ruminant animals and, crucially, between ruminant dairy and carcass fats, due to biosynthetic differences between the major fatty acids^41,42^. Thus, combined chemical and isotopic evidence for dairy lipids in Namaqualand pottery would unambiguously confirm the presence of domestic animals, and thus herders, even when their remains are absent or unidentifiable.

### Namaqualand

Namaqualand, located in north-western South Africa (Fig. 1), covers some 50,000 km^2^. It is bounded by the Atlantic Ocean to the west, the Kamiesberg Mountains ~ 100 km to the east, the Oliphants River to the south and the Orange River to the north. Namaqualand falls within the winter rainfall zone (WRZ) of southwestern Africa, which stretches from southwestern Namibia to Cape Agulhas and extends inland to the western margin of the Great Escarpment^43^. The WRZ receives more than 65% of its annual rainfall during the austral winter, but precipitation varies between 50 and 350 mm per year^44^. In Namaqualand the average annual rainfall ranges from 150 mm in the south to less than 50 mm near the Orange River^45^. This rainfall pattern is caused by the cold Benguela current and the upwelling of cold water from the Atlantic Ocean^46^. Due to this, Namaqualand is classified as a cold desert with a mean annual temperature of 16.8 °C^47^ but there are marked seasonal and diurnal extremes which may range from − 6 to 35 °C^45^. Palaeoclimatic records suggest some climatic fluctuations over the last 2000 years, particularly during the Medieval Warm Epoch (900 to 1300 AD) and the Little Ice Age (1300 to 1850 AD)^48–57^.

The unique flora of Namaqualand is characterized by remarkable plant diversity^58^, particularly among leaf succulent species^45^, which form the dominant component of vegetation cover (i.e. erect succulent shrubs). Whilst C_3_ plant species dominate in the region, Crassulacean Acid Metabolism (CAM) is widespread among leaf succulent species in Namaqualand, particularly within the dominant Mesembryanthemaceae^59,60^.

### Location and description of archaeological sites used in the current study

To help unravel the economic choices of LSA herders and foragers, we selected four sites from Namaqualand, two containing domesticate remains (Jakkalsberg K, Jakkalsberg M)^61,62^ and a further two absent of domesticate remains but yielding pottery (SK2005/057A, SK2006/026)^61,63–65^, generally regarded as a proxy for herders^15,23,66,67^. These latter two sites fall within the right time period for sheep to be on the landscape and together the four sites span in time from the early first millennium AD (JKB M) to the later second millennium AD (JKB K, SK2005/057A, SK2006/026). Notably, both SK sites are located on the Atlantic coast whereas the two JKB sites are further inland (Fig. 1), situated along the south bank of the Orange River, South Africa’s largest river.

### Inland sites

JKB M is part of the Jakkalsberg complex of archaeological sites located near the Orange (Gariep) River^62^. It is an open-air site located by the Bloubos River, a tributary ~ 200 m from the Orange River (28°10′50.5″S, 16°53′13.0″E, Fig. 1). The site lies in a deep deflation hollow with the base being ~ 2 m below the surrounding eroded silt deposits^62^. JKB M has a poorly preserved faunal assemblage including hares and small/medium and small bovids^62^, as well as two sheep limb bones and one caprine tooth (Table 1)^61^. Radiocarbon analysis of ostrich eggshell from squares L32, L33, and M33 yielded a date of 1740 ± 75 years BP (GX-32760)^61^ that calibrates to between 137 and 534 AD at 2σ (Table 1). Orton^61^ considers this date to be too old for the presence of lugs^12,14^, which more often date to the second millennia AD, however, the small size of the ostrich eggshell beads is consistent with the date.

**Table 1:** Sites, radiocarbon dating material, radiocarbon dates (BP and cal AD), details of faunal assemblage, and references.

Site	Dating material	Radiocarbon date (BP)	Calibrated radiocarbon date 2σ (AD)	Faunal assemblage (MNI incl. when available)	References
Jakkalsberg M (JKB M)	Ostrich eggshell	1740 ± 75	137–534	Hare Bovids (sheep, small/medium, and small)	^61,62^
Jakkalsberg K (JKB K)	Bone	358 ± 26	1487–1643	Numerous fish Medium/large carnivore Equids Bovids (small/medium and small) 2 probable sheep bones	^61,62^
SK2005/057A	Charcoal	400 ± 22	1455–1625	4564 shellfish Fish 251 Cape rock lobster Micromammal Snake Tortoise Bovids (3 steenbok, 1 cow, 1 small/medium bovid, and 2 small) 2 small/medium canids 4 small carnivores 1 Felis sp.	^61,63^
SK2006/026	Bone	370 ± 45 420 ± 45 430 ± 45	1460–1642 1446–1629 1439–1628	2056 shellfish 123 bovids (likely all springbok) 225 Cape rock lobster 1 seal small/medium mammals 2 fish 1 angulate tortoise 1 snake 1 small bird	^64,65^

JKB K, an open-air site located on the south bank of the Orange River (28°10′56.1″S, 16°52′55.9″E; Fig. 1), lies in a small, deflated area on the inland side of a vegetated riverine dune cordon^62^. The faunal assemblage consists primarily of fish^62^ with poorly preserved mammal remains including small and medium sized bovids and two faunal limb bones identified as ‘probable sheep’^61^. Such undiagnostic sheep elements are difficult to differentiate from other bovids of the same size^7,17^ and to our knowledge their attributions have not been confirmed by aDNA or Zooarchaeology by Mass Spectrometry (ZooMS). Although the fish remains at this site have not been identified, Orton and Halkett^68^ note that *Labeobarbus aeneus* (smallmouth yellowfish), *Labeo capensis* (Orange River mudfish), *Clarias gariepinus* (sharptooth catfish), *Labeobarbus kimberleyensis* (largemouth yellowfish), and *Labeo umbratus* (mud mullet) are the most economically valuable in this area^69^ and are thus likely species in LSA faunal assemblages along the lower Orange River. Radiocarbon analysis of steenbok bone from square K60 yielded a date of 358 ± 26 years BP (OxA-24528)^61^, calibrated to 1487 AD and 1643 AD at 2σ (Table 1).

### Coastal sites

SK2005/057A is a single occupation open-air site, situated on top of a prominent vegetated sand dune 1.8 km south of the Buffels River estuary and 780 m from the Atlantic Ocean (29°41′19.0″S, 17°03′48.7″E; Fig. 1). Although a small part of the site was disturbed, the majority was in pristine condition beneath 30 cm of sterile dune sand^61^. The faunal assemblage includes fish, shellfish, micromammal, snake, tortoise, *Jasus lalandii* (Cape rock lobster), *Felis libyca* (wild cat), small-medium canid (fox or jackal), *Ictonyx striatus* (striped polecat), *Cynictis penicillata* (yellow mongoose), *Raphicerus campestris* (steenbok), *Bos taurus* (domestic cattle), and unidentified small through large sized bovids (Table 1)^61,63^. Radiocarbon analysis of charcoal yielded a date of 400 ± 22 years BP (OxA-22981)^61^ that calibrates to between 1456 and 1625 AD at 2σ (Table 1).

SK2006/026 is a single occupation, springbok mass-kill site located 1.0 km south of the Buffels River estuary and 800 m from the Atlantic Ocean (29.6825°S, 17.06302778°E, Fig. 1). A total of 51.25 m^2^ was excavated stratigraphically down to sterile sand, with three archaeological layers (Surface, Lower, and Lower 2) and one sterile lens identified^65^. The faunal assemblage includes numerous highly fragmented *Antidorcas marsupialis* (springbok), as well as an *Arctocephalus pusillus* (cape fur seal), small/medium mammals, fish, shellfish, Cape rock lobster, *Chersina angulata* (angulate tortoise), snakes, and small birds (Table 1)^65^. Radiocarbon analyses of springbok bone from the surface and base layers yielded dates of 370 ± 45 years BP (Pta-9124), 420 ± 45 years BP (Pta-9105), and 430 ± 45 years BP (Pta-9099) respectively^64,65^. The radiocarbon dates have been calibrated to between 1460–1642 AD, 1446–1629 AD, and 1439–1628 AD at 2σ (Table 1).

Radiocarbon dates were calibrated using OxCal 4.4^70^ and the ShCal20^25^ curve.

## Results

Lipid analysis and interpretations were performed using established protocols described in detail in earlier publications^41,71^. A total of 106 potsherds and one surface or ‘burnt-on’ residue, were analysed from the four sites (Tables 2 and 3) with 78 yielding interpretable lipid profiles. The recovery rate was excellent at 74% overall, but varied between sites, being particularly high at JKB M at 91% (Table 2). The overall mean lipid concentration from the sherds was 1.5 mg g^−1^, with a maximum lipid concentration of 13.3 mg g^−1^ for sherd NAM77 (Table 3). Several of the potsherds contained very high concentrations of lipids (e.g., NAM41, 4.3 mg g^−1^; NAM75B, 10.7 mg g^−1^; NAM103A, 7.0 mg g^−1^ and NAM103B, 10.1 mg g^−1^) demonstrating excellent preservation (Table 3) and suggesting sustained use of these vessels in processing high lipid-yielding commodities.

**Table 2:** Number of sherds analysed, number of lipid-yielding sherds, % lipid recovery, and mean lipid concentration (mg g^−1^), by site.

	Number of sherds analysed	Lipid yielding sherds	% Lipid recovery	Mean lipid concentration (mg g^−1^)
SK2005/057	18	14	78	0.8
SK2006/026	29	19	66	1.5
Jakkalsberg M (JKB M)	23	21	91	2.4
Jakkalsberg K (JKB K)	36	24	67	1.4
Total	106	78	74%

**Table 3:** Lab/sample number, site, object number, lipid concentration (μg g^−1^), δ^13^C and Δ^13^C values, marine biomarkers present (n/a = not analysed and n/d = not determined) and attribution of residues.

Laboratory number	Site	Object number	Lipid concentration (µg g^−1^)	δ^13^C_16:0_	δ^13^C_18:0_	∆^13^C	Marine biomarkers	Attribution
NAM19	SK2005/057	AA21	740.8	− 21.9	− 21.9	0.0	LC APAAs, IPAs	Marine
NAM20	SK2005/057	AB21	326.0	− 21.7	− 21.7	0.0	n/a	Ruminant/non-ruminant adipose
NAM21	SK2005/057	AB22	2719.4	− 22.2	− 22.3	− 0.1	LC APAAs, IPAs	Marine
NAM22	SK2005/057	AB23	432.9	− 21.9	− 22.3	− 0.4	LC APAAs	Marine
NAM23	SK2005/057	AC22	3609.0	− 22.2	− 21.9	0.3	LC APAAs, IPAs	Marine
NAM24A	SK2005/057	W13	50.3	− 22.5	− 23.9	− 1.4	LC APAAs	Marine
NAM24ARes	SK2005/057	W13	1977.6	− 21.6	− 23.1	− 1.5	n/a	Ruminant adipose
NAM24C	SK2005/057	W13	209.5	− 22.4	− 22.5	− 0.1	LC APAAs	Marine
NAM25	SK2005/057	W14	192.8	− 22.5	− 22.0	0.5	n.d (C_18_ APAAs only)	Non-ruminant adipose
NAM29	SK2005/057	Y14	1813.9	− 22.0	− 21.9	0.1	LC APAAs	Marine
NAM30	SK2005/057	Y15	493.4	− 22.0	− 21.8	0.1	LC APAAs	Marine
NAM31	SK2005/057	Y21	53.3	− 22.1	− 22.3	− 0.2	LC APAAs	Marine
NAM32	SK2005/057	Y23	251.9	− 22.4	− 22.6	− 0.2	n/a	Ruminant/non-ruminant adipose
NAM33A	SK2005/057	Z16	164.2	− 21.3	− 21.0	0.3	n/a	Ruminant/non-ruminant adipose
NAM33B	SK2005/057	Z16	159.5	− 21.9	− 22.2	− 0.3	n/a	Ruminant/non-ruminant adipose
NAM34	SK2006/026	D35	3443.4	− 24.1	− 26.2	− 2.0	n/a	Ruminant adipose
NAM35	SK2006/026	F24A	744.9	− 24.6	− 26.5	− 1.8	IPAs	Marine
NAM38	SK2006/026	G29B	1709.9	− 24.7	− 26.2	− 1.6	LC APAAs	Marine
NAM39	SK2006/026	G30B	1718.9	− 24.6	− 26.7	− 2.1	LC APAAs, IPAs	Marine
NAM40	SK2006/026	G30C	1751.5	− 24.1	− 26.5	− 2.3	LC APAAs, IPAs	Marine
NAM41	SK2006/026	G30D	4347.9	− 23.8	− 26.8	− 3.0	LC APAAs	Marine
NAM43A	SK2006/026	H25C	482.4	− 23.7	− 25.7	− 2.1	LC APAAs	Marine
NAM43B	SK2006/026	H25C	724.3	− 24.6	− 26.6	− 2.0	LC APAAs	Marine
NAM44	SK2006/026	H27C	582.8	− 25.5	− 26.4	− 0.9	n.d (C_18_ APAAs only)	Ruminant adipose
NAM48	SK2006/026	H28B	2035.8	− 23.1	− 22.1	1.0	LC APAAs	Marine
NAM49A	SK2006/026	H29CS	3048.0	− 24.9	− 26.7	− 1.7	LC APAAs	Marine
NAM50	SK2006/026	I24A	1559.0	− 24.4	− 27.1	− 2.6	LC APAAs	Marine
NAM51	SK2006/026	I29A	727.7	− 23.9	− 22.7	1.2	LC APAAs, IPAs	Marine
NAM52	SK2006/026	I30A	298.3	− 21.8	− 20.2	1.6	n.d	Non-ruminant adipose
NAM53	SK2006/026	I30B	2395.6	− 23.6	− 22.5	1.1	LC APAAs, IPAs	Marine
NAM54	SK2006/026	J23C	1131.1	− 24.5	− 26.4	− 1.9	LC APAAs	Marine
NAM56	SK2006/026	K28D	374.9	− 22.2	− 24.2	− 2.0	n/a	Ruminant adipose
NAM57	SK2006/026	L27B	1615.2	− 23.4	− 25.0	− 1.6	n/a	Ruminant adipose
NAM59	SK2006/026	L28D	499.2	− 21.9	− 24.1	− 2.1	n/a	Ruminant adipose
NAM61	JKB M	M35	577.1	− 23.6	− 25.5	− 1.9	n.d (C_18_ APAAs only)	Ruminant adipose
NAM62	JKB M	R24	235.9	− 25.0	− 26.2	− 1.2	n.d	Ruminant adipose
NAM63	JKB M	P24	380.6	− 25.0	− 26.3	− 1.3	n.d	Ruminant adipose
NAM64	JKB M	O38	593.4	− 20.0	− 21.4	− 1.4	n.d (C_18_ APAAs only)	Ruminant adipose
NAM65	JKB M	O40	1056.1	− 20.6	− 21.6	− 1.0	n.d (C_18_ APAAs only)	Ruminant adipose
NAM66	JKB M	O39	525.1	− 20.7	− 21.5	− 0.8	n.d (C_18_ APAAs only)	Ruminant/non-ruminant adipose
NAM67	JKB M	Q24	343.8	− 24.3	− 26.3	− 1.9	n.d	Ruminant adipose
NAM68	JKB M	S23	3464.3	− 24.9	− 25.9	− 1.0	n.d (C_18_ APAAs only)	Ruminant adipose
NAM69	JKB M	P39	2798.1	− 20.6	− 21.2	− 0.6	n.d (C_18_ APAAs only)	Ruminant/non-ruminant adipose
NAM70	JKB M	O24	313.7	− 24.9	− 26.3	− 1.4	n.d	Ruminant adipose
NAM72	JKB M	M34	1131.9	− 23.9	− 25.5	− 1.6	n.d (C_18_ APAAs only)	Ruminant adipose
NAM73A	JKB M	P37	2672.7	− 23.0	− 24.6	− 1.6	n.d (C_18_ APAAs only)	Ruminant adipose
NAM73B	JKB M	P37	1222.6	− 20.3	− 21.4	− 1.0	n.d (C_18_ APAAs only)	Ruminant adipose
NAM75A	JKB M	O36	2102.2	− 23.9	− 25.0	− 1.1	n.d (C_18_ APAAs only)	Ruminant adipose
NAM75B	JKB M	O36	10,734.3	− 22.7	− 24.4	− 1.7	n.d (C_18_ APAAs only)	Ruminant adipose
NAM76	JKB M	K29	2200.7	− 22.4	− 26.0	− 3.6	n/a	Ruminant dairy
NAM77	JKB M	P36	13,310.5	− 23.3	− 24.5	− 1.2	n/a	Ruminant adipose
NAM78	JKB M	N36	2438.8	− 24.3	− 25.3	− 0.9	n/a	Ruminant adipose
NAM79	JKB M	P35	4155.7	− 23.1	− 25.0	− 1.9	n/a	Ruminant adipose
NAM80A	JKB M	N35	674.7	− 23.9	− 25.7	− 1.8	n/a	Ruminant adipose
NAM80B	JKB M	N35	159.3	− 24.3	− 26.3	− 2.1	n/a	Ruminant adipose
NAM81	JKB K	J60	1260.8	− 24.4	− 26.2	− 1.8	n.d (C_18_ APAAs only)	Ruminant adipose
NAM82	JKB K	G62	183.6	− 21.8	− 26.4	− 4.6	n.d	Ruminant dairy
NAM83	JKB K	I56	255.6	− 24.0	− 25.4	− 1.5	n.d	Ruminant adipose
NAM84A	JKB K	G58	677.0	− 22.9	− 25.3	− 2.4	n/a	Ruminant adipose
NAM84B	JKB K	G58	1636.9	− 22.7	− 24.3	− 1.6	n/a	Ruminant adipose
NAM85	JKB K	K60	2445.4	− 24.1	− 26.1	− 2.0	n.d (C_18_ APAAs only)	Ruminant adipose
NAM86	JKB K	J57	564.2	− 23.4	− 25.6	− 2.2	n.d	Ruminant adipose
NAM87	JKB K	I55	421.1	− 23.7	− 25.4	− 1.7	n.d	Ruminant adipose
NAM88	JKB K	K61	1249.9	− 23.2	− 25.9	− 2.7	n.d	Ruminant adipose
NAM89	JKB K	R47	1074.5	− 23.8	− 26.0	− 2.2	n.d (C_18_ APAAs only)	Ruminant adipose
NAM90	JKB K	R60	88.6	− 24.0	− 26.3	− 2.3	n.d (C_18_ APAAs only)	Ruminant adipose
NAM94	JKB K	K62	263.9	− 23.9	− 25.7	− 1.8	n.d	Ruminant adipose
NAM96	JKB K	I57	109.5	− 24.2	− 25.5	− 1.3	n/a	Ruminant adipose
NAM97	JKB K	K63	618.9	− 24.6	− 26.5	− 1.9	n/a	Ruminant adipose
NAM99	JKB K	I59	737.4	− 23.9	− 26.0	− 2.1	n/a	Ruminant adipose
NAM101	JKB K	R54	107.5	− 24.2	− 25.2	− 0.9	n/a	Ruminant adipose
NAM102A	JKB K	AREA A	1005.9	− 23.7	− 25.3	− 1.6	n/a	Ruminant adipose
NAM103A	JKB K	AREA B	7029.0	− 26.0	− 27.3	− 1.3	n/a	Ruminant adipose
NAM103B	JKB K	AREA B	10,141.3	− 19.7	− 24.9	− 5.2	n/a	Ruminant dairy
NAM105	JKB K	S53	2826.2	− 23.5	− 25.9	− 2.5	n/a	Ruminant adipose
NAM107	JKB K	O56	240.4	− 21.1	− 23.8	− 2.6	n/a	Ruminant adipose
NAM108	JKB K	M62	109.1	− 20.8	− 22.1	− 1.3	n/a	Ruminant adipose
NAM111	JKB K	Q58	759.2	− 25.0	− 26.6	− 1.6	n/a	Ruminant adipose
NAM112	JKB K	F59	146.5	− 22.5	− 24.4	− 1.9	n/a	Ruminant adipose

**Figure 3:**
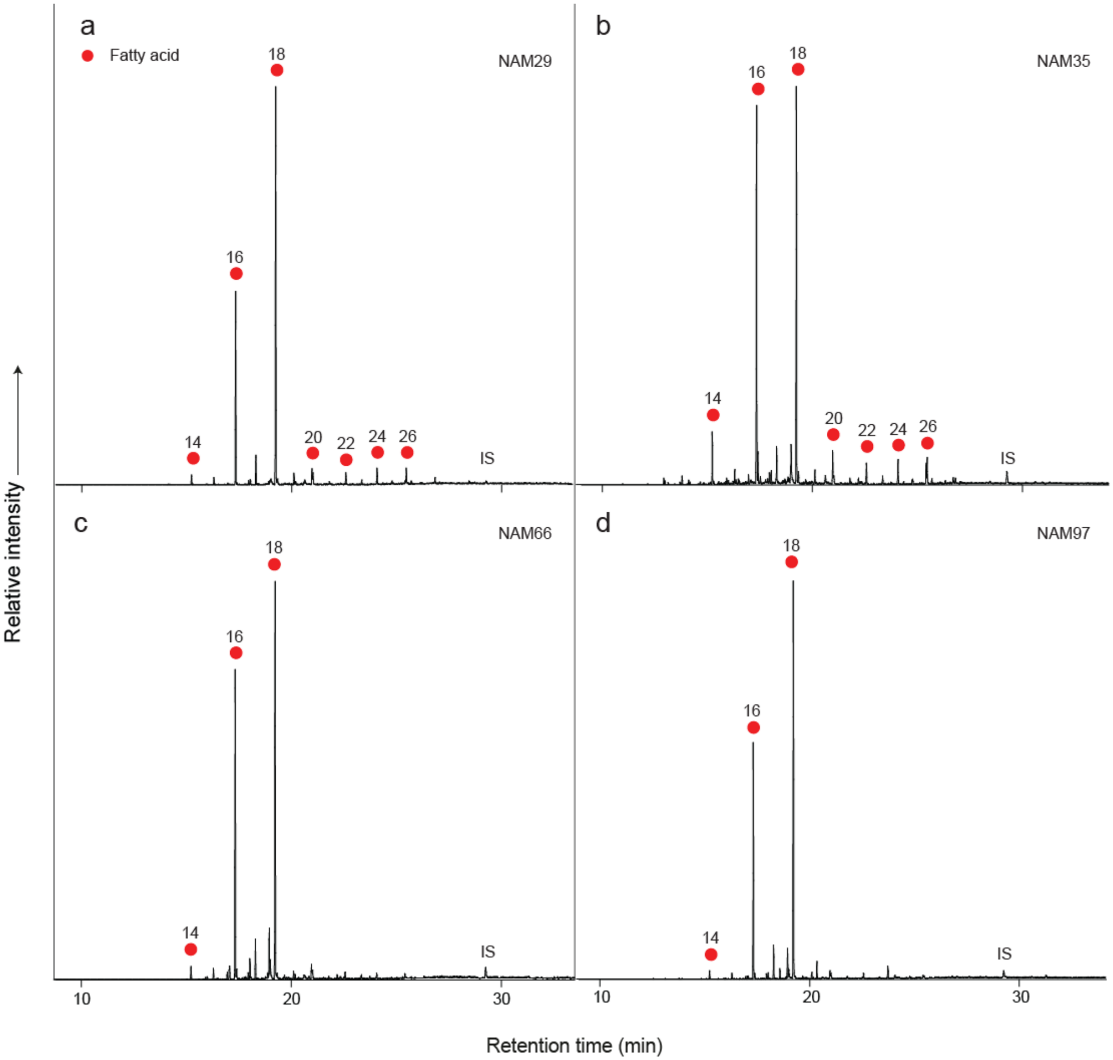
Partial gas chromatograms of acid-extracted FAMEs showing typical degraded animal fat lipid profiles from sites (**a**). SK2005/057, (**b**). SK2006/026, (**c**). JKB M and (**d**). JKB K. Red circles, *n*-alkanoic acids (fatty acids, FA), IS, internal standard, C_34_ *n*-tetratriacontane. Number denotes carbon chain length.

Analysis of the total lipid extracts (*n* = 78) from the four sites, using gas chromatography (GC) and gas chromatography-mass spectrometry (GC–MS), demonstrated they were dominated by the free fatty acids, palmitic (C_16_) and stearic (C_18_) acids, typical of a degraded animal fat (Fig. 3)^72,73^.

**Figure 4:**
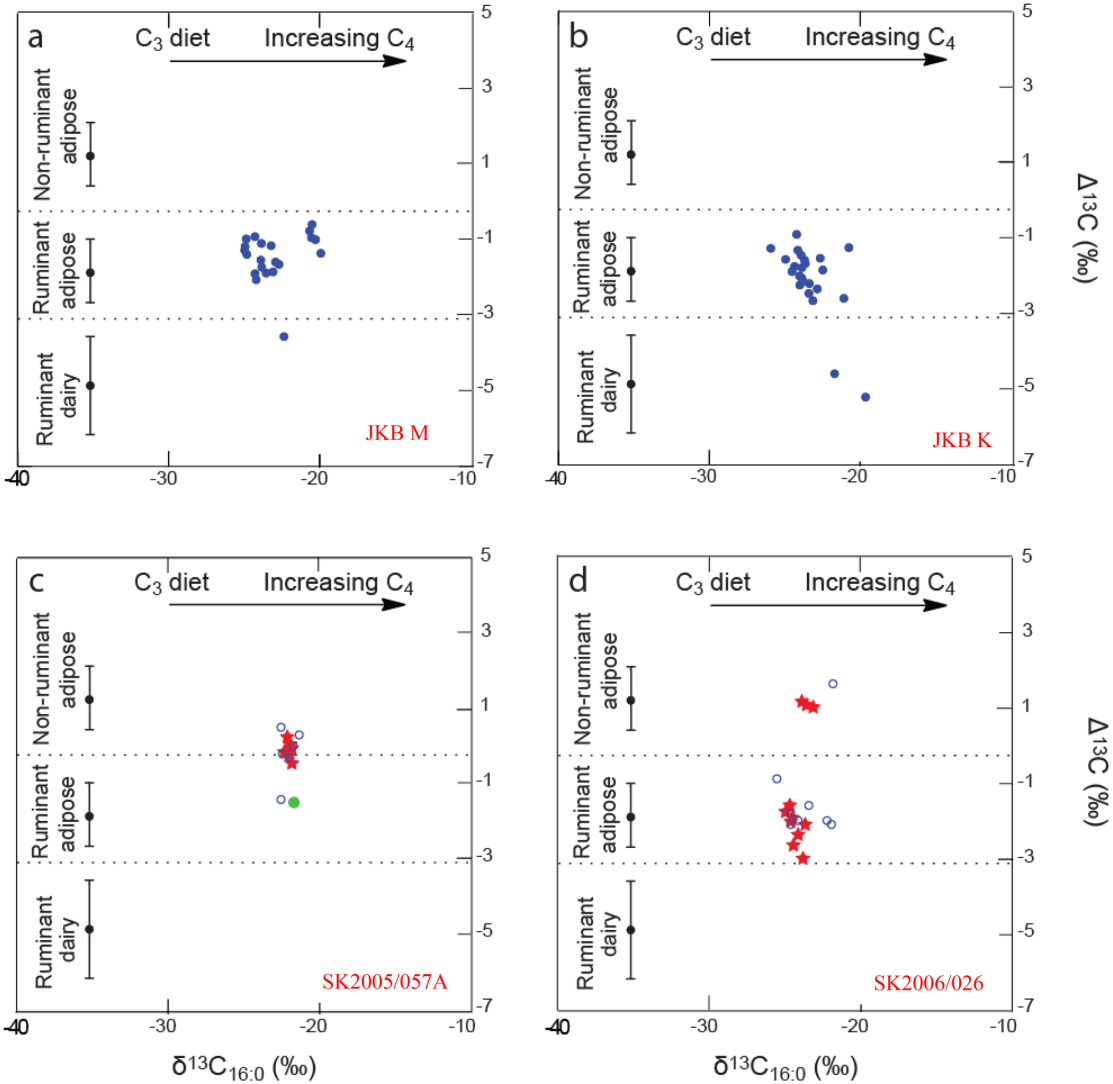
Graphs showing the Δ^13^C (δ^13^C_18:0_–δ^13^C_16:0_) values from the Namaqualand potsherds by individual site. Datapoints shown as blue (filled circles in **a** and **b** and unfilled circles in **c** and **d**) indicates the processing of terrestrial animal fats and red stars indicate where APAAs of carbon chain length C_18_–C_22_ and isoprenoid acids (indicating marine resource processing) were also observed in the residue. The green dot indicates the external residue analysed. The ranges shown here represent the mean ± 1 s.d. of the Δ^13^C values for a global database comprising modern reference animal fats from Africa^75^, UK (animals raised on a pure C_3_ diet)^41^, Kazakhstan^76^, Switzerland^77^, and the Near East^78^ published elsewhere.

These lipid extracts underwent gas chromatography-combustion-isotope ratio mass spectrometry (GC-C-IRMS) analyses (Fig. 4, Table 3) to determine the δ^13^C values of the major fatty acids, C_16:0_ and C_18:0_, which reflect their biosynthetic and dietary origin, and ascertain the source of the lipids extracted^41,74,75^.

Across the four sites, the δ^13^C_16:0_ values of the fatty acids range from − 25.5 to − 19.7‰ and the δ^13^C_18:0_ values range from − 27.3 to − 20.2‰ (Table 3). This relatively wide range of values could be interpreted as resulting from the processing of carcass products from terrestrial animals subsisting on diets of mostly C_3_ and, possibly, minor amounts of C_4_ or CAM plants. As noted, CAM plants are commonplace in the region. A large-scale study (*n* = 103) on leaf tissues of vascular plant species from the arid Richtersveld of northern Namaqualand revealed two distinct arrays of δ^13^C values^60^. The C_3_ species analysed had values ranging from -13 to -21‰, with the largest number of species falling in the − 17 to − 18‰ range, in contrast to values for the CAM (mainly succulent) flora where the range is − 4 to − 8‰. Alternatively, and bearing in mind the proximity of SK2005/057A and SK2006/026 to the Atlantic Ocean and JKB M and JKB K to the Orange River, the somewhat enriched δ^13^C values could be indicative of mixtures between terrestrial and marine/freshwater commodities^39,79,80^.

Consequently, 50 lipid-yielding samples (SK2005/057A, *n* = 10; SK2006/026, *n* = 15; JKB M, *n* = 15 and JKB K, *n* = 10, see Table 3) were sub-sampled from the original 78 for analysis by GC–MS in selected ion monitoring (SIM) mode to check for the presence of freshwater/marine biomarkers, such as vicinal dihydroxy acid (DHYAs) and *ω*-(*o*-alkylphenyl) alkanoic acids (APAAs), which would confirm the presence of precursor long-chain mono- and polyunsaturated fats, respectively, abundant in fresh marine/aquatic fats such as fish, marine mammal, shellfish, and crustaceans. In addition, a third class of biomarker, isoprenoid fatty acids (IFAs), comprising 4,8,12-trimethyltridecanoic acid (TMTD), 3,7,11,15 tetramethylhexadecanoic acid (phytanic acid), and 2,6,10,14-tetramethylpentadecanoic acid (pristanic acid), important biomarkers in the detection of marine food processing^39,81,82^, were investigated. However, it should be noted that phytanic acid is also found in low concentrations in terrestrial fats such as milk^83–85^ and thus can only be interpreted as a marine biomarker when found in association with TMTD (which only occurs in the marine environment) and pristanic acid^84^. The presence and combinations of these biomarkers are routinely used to detect marine product processing^39,80,82,86,87^. Notably, here, their identification in specific vessels demonstrates that the lipid profiles fall into two distinct categories: marine resources (fish, shellfish, Cape rock lobster and seal processing) at coastal sites (SK2005/057A and SK2006/026) or terrestrial products (meat and milk) at inland sites (JKB M and JKB K).

### Fish, shellfish, cape rock lobster and seal processing

Notably, at SK2005/057A, a coastal site, seven of the ten vessels (70%) which underwent SIM contained C_18_, C_20_ and C_22_ APAAs and a further two sherds produced the C_18_ and C_20_ APAAs, providing unambiguous evidence for the processing of marine products in virtually all vessels tested, possibly in conjunction with animal carcass fats. Two of these vessels also included the 4,8,12-TMTD, pristanic acid and phytanic acid with one only containing the 4,8,12-TMTD (Table 3 and Fig. 4c), further strengthening these attributions. At SK2006/026, nine of nineteen (47%) vessels contained C_18_, C_20_ and C_22_ APAAs and two (10%) included the C_18_ and C_20_ APAAs (Table 3 and Fig. 4d). Also present in five of the SK2006 vessels were 4,8,12-TMTD and phytanic acid. This suggests that marine products were processed in at least two thirds of vessels analysed at the two coastal sites. Since APAAs are only formed at high temperatures (at *c*. 270 °C), from the protracted heating of polyunsaturated fatty acids, their presence suggests prolonged boiling of either fish, shellfish, rock lobster or sea mammals in pots at SK2005/057A and SK2006/026, confirmed by extremely high lipid concentrations in many of the vessels. The absence of detectable marine biomarkers in the remaining vessels that underwent SIM does not preclude the processing of marine fats, since they may not have survived or, possibly, were processed under conditions not conductive to their formation.

### Meat and milk

In contrast, at the Orange River inland sites of JKB K and JKB M, there is little evidence for the exploitation of freshwater resources, with eleven (of fifteen, 73%) and four (of ten, 40%) vessels, respectively, only yielding the C_18_ APAAs. At both sites, the vessels appear to predominantly be used to process ruminant adipose products, confirmed by the presence of sheep and small bovid bone at both sites (Table 1 and Fig. 4a,b). Notably, the δ^13^C values of the ruminant lipids from these sites are of C_3_ origin, albeit somewhat enriched, suggesting they originate from grazing species (sheep or other bovids) subsisting on mainly grasses (and the possible addition of minor amounts of C_4_ or CAM plants), rather than browsers exploiting enriched (CAM) plant succulent species. It is also possible that, should the vessels have been used occasionally for dedicated plant processing of C_4_/CAM plants, then fatty acids derived from these could contribute more enriched δ^13^C values to the overall fatty acid signature of the C_16:0_ and C_18:0_ fatty acids. These results confirm the importance of livestock and/or hunted small bovids, as a meat source over at least the span of the last 2000 years.

Most notably, evidence for dairy processing is present at both sites. At JKB M, one vessel plots within the ruminant dairy region (NAM76, Δ^13^C value of – 3.6‰, Fig. 4a) and the remainder (*n* = 18) plot within the ruminant adipose region, save for two sherds (NAM66, Δ^13^C = − 0.8‰) and NAM69 (NAM69, Δ^13^C = − 0.6‰) which plot between the ruminant and non-ruminant regions. At JKB K two vessels, NAM82 and NAM103B, plot within the ruminant dairy region (Δ^13^C values of − 4.6 and − 5.2‰, respectively) with the rest plotting within the ruminant adipose region (Fig. 4b, Table 3).

## Discussion and conclusions

Here, organic residue analysis of archaeological potsherds from Namaqualand provides the first direct chemical evidence for people processing milk and/or milk products in ceramic pottery in western South Africa during the LSA. Three of the 78 lipid-yielding sherds, originating from inland sites which produced sheep bones, contained dairy lipids. These results bear out the ethnohistoric accounts, which suggest milk was usually stored in organic containers but that pots were occasionally used to cook milk, often together with other foodstuffs (see Refs.^25,28,33–36^ for examples). Given that the milk from their animals is thought to be the staple feature of LSA herder diets^25^, at least in the second millennium AD, it could be hypothesised that dairy processing at these sites may well be under-represented, largely taking place in organic vessels such as calabashes and leather bags, particularly bearing in mind its greater prevalence at sites in Lesotho^40^. Similarly, in a recent study^88^ on modern-day Samburu pottery from Kenya, combining lipid analysis and ethnographic information, the Samburu pottery lipid residues were found to reflect the functional and ideological suitability of ceramics for processing only certain types of food (meat/fat/bones), despite an overall reliance on milk in their diet^88^.

The lipid analysis has thus confirmed both the antiquity (from the earliest introduction of sheep) and longevity (to the historic period) of the use of milk products as part of a herd management strategy (Table 3). The high lipid concentrations in two of these dairy sherds, (one from each site, i.e., NAM76, 2.2 mg g^−1^ and NAM103b, 10.1 mg g^−1^; Table 3) suggests the vessels saw sustained use, probably in boiling or cooking. Furthermore, two of these fall well within the dairy range, indicating that it is unlikely that people were using these pots to cook milk in combination with other foodstuffs, i.e. meat, as some ethnohistoric^36^ accounts document. The processing of milk and plants together^21^, however, cannot be ruled out as low lipid-yielding plants may well be ‘swamped’ by milk fats, which contain several orders of magnitude higher lipid concentrations than the lipid concentrations of plant-based foods^89,90^.

The identification of marine product processing in most vessels at the two coastal sites (and absence of evidence for dairy processing), SK2005/057A and SK2006/026, are in good agreement with those obtained from the pre-colonial site Kasteelberg De^21^ where spouted ware vessels were used to process marine-derived animal products, despite sheep bones being abundant at the site. However, it should be noted that the occupation of Kasteelberg De was earlier (eighth–eleventh centuries AD)^39^ compared to the coastal sites analyzed here (fifteenth–seventeenth centuries AD)^61,65^, and are located about 600 km apart. Their faunal assemblages also differ, e.g., seals at KBD versus Cape rock lobster at SK2005/057A and SK2006/026, possibly reflecting the availability of differing resources at each site.

At SK2005/057A the pottery lipids ultimately reflect the mixed processing of terrestrial and marine animal products (Fig. 4). Since this is a single occupation open-air site, the results suggest that the people occupying the site may have carried their pottery with them as they moved around the landscape and likely used and reused their pots to process whichever resources were available at different locations and times of the year. Notably, the presence of APAAs in the (absorbed) lipid residues from vessel NAM24 suggests the processing of marine products during the lifetime use of the vessel. Conversely, these are absent from the surface residue which was found to comprise ruminant adipose products, thus representing the final foodstuffs cooked within this pot^91^.

**Figure 5:**
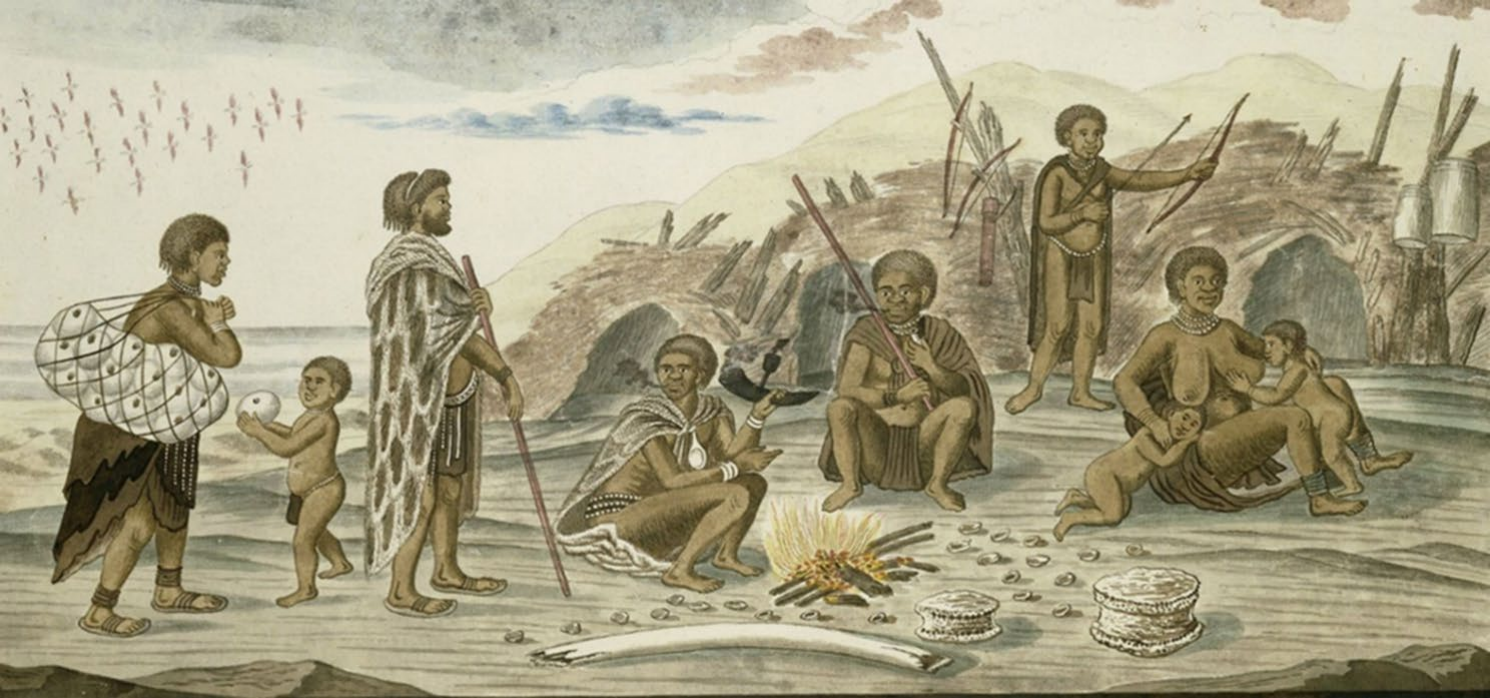
‘*Homestead of the so-called Strandlopers (San or Khoikhoi) just north of the Orange River’s mouth*’, showing the roasting of what appears to be shellfish over the fire. Attributed to Robert Jacob Gordon, 1779. Adapted from https://www.rijksmuseum.nl/nl/collectie/RP-T-1914-17-91.

Nonetheless, the dominance of shellfish and Cape rock lobster in the faunal assemblage suggests that two thirds of vessels at the site containing marine biomarkers were used in their processing. Along with several shellfish and vertebrate species, the Cape rock lobster (*Jasus lalandii*) was an important marine resource for people during the precolonial past along the Western Cape coast^92^. However, although there are no ethnohistoric or ethnographic accounts (to our knowledge) for how people specifically cooked either, an image attributed to Robert Jacob Gordon (Fig. 5) does show a group of people just north of the Orange River’s mouth, roasting what appears to be shellfish over the fire^22^ thus it may be more likely that people boiled Cape rock lobster in pots and roasted shellfish over fires. Alternatively, and since it is likely that people carried their pots from site to site instead of making new ones at each location, the marine signal seen in the pots at SK2005/057A may originate from rendering of some seal fats even though there are none in the assemblage (Table 1). Seal fat processing would likely produce the high lipid concentrations seen in many of the vessels from both sites (see Table 3), which do suggest the intensive and/or specialized processing of marine products. The boiling of seal fat/meat is well-documented ethnohistorically, for example, van Riebeeck^3,26^ noted that some Khoekhoen groups used seal oil for body adornment as well as to add fat to their diet.

In contrast, the faunal assemblage at SK2006/026 may suggest distinct uses for the vessels. Marine products processed in the vessels likely again comprised Cape rock lobster and/or shellfish, which dominate the faunal assemblage, although one seal bone is also present (Table 1). However, the faunal assemblage is unusual in that it includes a large number of one species, namely springbok antelope (a ruminant), indicating that it was a springbok kill site. Five of the six vessels which did not include marine biomarkers from SK2006/026 plot in the ruminant carcass region and contain generally high lipid concentrations, hinting that they may have been used for large-scale processing of springbok meat, although some minor marine input cannot be discounted.

A taphonomic analysis of the springbok assemblage^25^, found that the long bones were highly fragmented, ranging from 24.3 to 40.9 mm in length and over half (54%) displayed spiral fractures, indicating they were broken when fresh. This pattern is consistent with intentional fracturing to boil bones to extract marrow/grease^65^. While it is unknown if grease was being produced for body adornment^26^, to supplement a generally fat-lean diet^27^, or to extract all available nutrients during a nutritionally lean time, the demographic profile of the springbok indicates that the site was occupied during a drought^57,64,65^. The zooarchaeological results are thus in good agreement with stable light isotope analysis of the juvenile springbok antelope teeth, which identified a trend towards high δ^15^N values during tooth formation^57^ with the most enriched numbers occurring closer to the animals’ death. These results may suggest vessels did indeed serve distinct purposes, possibly indicating that people were intensively processing springbok antelope and Cape rock lobster and/or seal in specific pots or at least, in some vessels, intensive processing of springbok swamped out any other lipid signal.

In summary, the combination of ethnohistoric accounts, faunal information, and lipid analysis has provided valuable information on the economic strategies of LSA herders and foragers across the unpredictable environment of the northwestern Cape of South Africa. Our results confirm the extensive processing of marine products by foragers at coastal sites and the importance of both meat and milk products to early stockkeepers.

## Methods

A total of 106 sherds from the four sites were selected for analysis (see Table 2). At Jakkalsberg M, we selected 23 undiagnostic body sherds from the 202 potsherds (including at least 6 rim sherds) recovered from the site. Nineteen sherds were decorated with incised horizontal lines^61,62^ and three lugs and two small bosses were present. A total of 798 sherds were excavated from Jakkalsberg K, comprising 24 undecorated rim sherds, two lugs, and one sherd possibly from the base of a spout^61^. Of these, we selected 36 undiagnostic body sherds for analysis. At SK2005/057A, 86 potsherds were recovered, including a single rim fragment with two rows of impressions, a plain rim sherd, and a cluster of very fine-grained and thinner-walled sherds^61^. Here we selected 18 undiagnostic body sherds for analysis. Finally, 160 potsherds were recovered from the site SK2006/026, including five rim sherds, one of which was decorated with two parallel rows of impressed dots. There were no identifiable lugs in the ceramic assemblage, although one body sherd appears to have been reinforced internally, perhaps forming the edge of a lug^65^. A total of 29 undiagnostic body sherds were selected for analysis.

Lipid analysis and interpretations were performed using established protocols described in detail in earlier publications^71^. Briefly, ~ 2 g of potsherd was sampled, and surfaces cleaned with a modelling drill to remove exogenous lipids. The cleaned sherd powder was crushed in a solvent-washed mortar and pestle and weighed into a furnaced culture tube (I). An internal standard was added (20 µg *n*-tetratriacontane; Sigma Aldrich Company Ltd) together with 5 mL of H_2_SO_4_/MeOH 2–4% (δ^13^C value measured) and the culture tubes were placed on a heating block for 1 h at 70 °C, mixing every 10 min. Once cooled, the methanolic acid was transferred to test tubes and centrifuged at 2500 rpm for 10 min. The supernatant was then decanted into another furnaced culture tube (II) and 2 mL of DCM extracted double distilled water was added.

In order to recover any lipids not fully solubilised by the methanol solution, 2 × 3 mL of *n*-hexane was added to the extracted potsherds contained in the original culture tubes, mixed well and transferred to culture tube II. The extraction was transferred to a clean, furnaced 3.5 mL vial and blown down to dryness. Following this, 2 × 2 mL *n*-hexane was added directly to the H_2_SO_4_/MeOH solution in culture tube II and whirlimixed to extract the remaining residues, then transferred to the 3.5 mL vials and blown down until a full vial of *n*-hexane remained. Aliquots of the TLE’s were derivatised using 20 µL BSTFA, excess BSTFA was removed under nitrogen and the derivatised TLE was dissolved in *n*-hexane prior to GC, GC–MS and GC-C-IRMS. Firstly, the samples underwent high-temperature gas chromatography using a gas chromatograph (GC) fitted with a high temperature non-polar column (DB1-HT; 100% dimethylpolysiloxane, 15 m × 0·32 mm i.d., 0.1 μm film thickness). The carrier gas was helium, and the temperature programme comprised a 50 °C isothermal followed by an increase to 350 °C at a rate of 10 °C min^−1^ followed by a 10 min isothermal.

A procedural blank (no sample) was prepared and analysed alongside every batch of samples. Further compound identification was accomplished using gas chromatography-mass spectrometry (GC–MS). FAMEs were then introduced by autosampler onto a GC–MS fitted with a non-polar column (100% dimethyl polysiloxane stationary phase: 60 m × 0.25 mm i.d., 0·1 μm film thickness). The instrument was a ThermoFinnigan single quadrupole TraceMS run in EI mode (electron energy 70 eV, scan time of 0·6 s). Samples were run in full scan mode (*m/z* 50–650) and the temperature programme comprised an isothermal hold at 50 °C for 2 min, ramping to 300 °C at 10 °C min^−1^, followed by an isothermal hold at 300 °C (15 min). The instrument was a ThermoFinnigan single quadrupole TraceMS run in EI mode (electron energy 70 eV, scan time of 0·6 s). Samples were run in full scan mode (*m/z* 50–650) and the temperature programme comprised an isothermal hold at 50 °C for 2 min, ramping to 300 °C at 10 °C min^−1^, followed by an isothermal hold at 300 °C (15 min). Data acquisition and processing were carried out using the HP Chemstation software (Rev. C.01.07 (27), Agilent Technologies) and Xcalibur software (version 3.0). Peaks were identified based on their mass spectra and gas chromatography (GC) retention times, by comparison with the NIST mass spectral library (version 2.0).

Selected lipid extracts were investigated using GC/MS-SIM for high-sensitivity detection of ω-(o-alkylphenyl) alkanoic acids (APAAs) and dihydroxy acids (DHFAs).

## Data Availability

All data produced in this study are included in the article.
